# Antimicrobial prophylaxis outside the operating theatre, an audit in a university hospital

**DOI:** 10.1186/s12879-017-2354-4

**Published:** 2017-04-21

**Authors:** Jan W. T. Deelen, Caroline E. Visser, Jan M. Prins, Reinier M. van Hest

**Affiliations:** 1 Academic Medical Center, Amsterdam, the Netherlands; 2 Pressent Address: Julius Center for Health Sciences and Primary Care, University Medical Center Utrecht, Huispostnr. STRATENUM 6.131, Postbus 85500, 3584 CG Utrecht, the Netherlands; 3 Department of Microbiology, Academic Medical Center, Amsterdam, the Netherlands; 4 Department of Internal Medicine, Academic Medical Center, Amsterdam, the Netherlands; 5 Department of Hospital Pharmacy, Academic Medical Center, Amsterdam, the Netherlands

**Keywords:** Antimicrobials, Antibiotics, Antibiotic stewardship, Prophylaxis, Point prevalence survey

## Abstract

**Background:**

The prophylactic use of antimicrobial agents to prevent infections in non-surgical situations has hardly been investigated. We investigate the extent, indications and appropriateness of antimicrobial prophylaxis given outside the operating room in a tertiary care hospital.

**Methods:**

Four point-prevalence surveys were conducted in which all inpatients on that day were screened for the use of prophylactic antimicrobials: medical prophylaxis, prophylaxis around non-surgical interventions and surgical prophylaxis given on the ward. The primary endpoint was the extent of prophylaxis relative to the total number of antimicrobial prescriptions. We also investigated per prescription the presence of a (local) protocol and adherence to these protocols.

**Results:**

We registered in total 1020 antimicrobial prescriptions, of which 317 (31.1%) were given as prophylaxis. 827/1020 were antibiotic prescriptions. Of these antibiotic prescriptions, 17.0% was medical prophylaxis, 2.7% prophylaxis around non-surgical interventions and 6.9% surgical prophylaxis administered on a ward. For medical antibiotic prophylaxis, a protocol was present in 125 of 141 prescriptions (88.7%); the protocol was followed in 118 cases (94.4%). For prophylaxis around non-surgical interventions and surgical prophylaxis on the wards, protocol presence and adherence rates were 59.1% and 92.3%, and 73.3% and 97.6% respectively. Of the 96 antiviral and 97 antifungal prescriptions, 42.7% and 57.8%, respectively, were medical prophylaxis, of which 95.1 and 96.3% were prescribed according to protocols respectively.

**Conclusions:**

Antimicrobial prophylaxis outside the operating theatre is responsible for a considerable part of total in-hospital antimicrobial use. For most prescriptions there was a protocol and adherence to the protocols was high. The main targets for improvement were prophylaxis around non-surgical interventions and surgical prophylaxis given on the ward.

## Background

Antimicrobial prophylaxis is an important part of antimicrobial use. Peri-operative antibiotic prophylaxis prevents up to 80% of surgical site infections [1]. The increasing use of high-dose chemotherapy and non-surgical invasive therapies (cardiology, gastro-enterology) has led to an increase of indications for medical antimicrobial prophylaxis: the prevention of infections in non-surgical situations. These indications have been described in reviews and guidelines [2–4]. They have, however, also been subjected to debate: low level evidence due to a lack of high quality clinical trials has led to different interpretations on what dosage of which antibiotic to use for what indication and for what duration. This carries a serious risk for suboptimal use.

In 2015, the Health Council of the Netherlands concluded that there is a lack of knowledge concerning the extent, indications, and evidence base of medical antimicrobial prophylaxis [5]. Published audits hardly seem to include any in-depth information on medical antimicrobial prophylaxis [6–8]. We suspect that it might be responsible for a considerable part of total in-hospital antimicrobial drug use.

In this study, we investigated in a point-prevalence survey the in-hospital use of antimicrobials for prophylaxis given outside the operating theatre in a tertiary care teaching hospital. The goals were threefold: to investigate the extent of prophylaxis, the indications for prophylaxis per medical subspecialty and the presence of protocols and guidelines and the adherence to these protocols. This will contribute to the identification of targets for intervention in line with the goals of antimicrobial stewardship.

## Methods

### Design and setting

The study was carried out in June 2015 in the Academic Medical Center in Amsterdam, a tertiary care hospital with 1002 hospital beds. The hematology department performs allogeneic and autologous stem cell transplantation, the surgery department performs complex oncological surgery (e.g. liver resections, Whipple procedures), and there is a kidney transplant unit (+/− 130 transplantations per year). An antimicrobial stewardship team is present, which monitors the use of restricted antimicrobial drugs. An infectious diseases specialist can be consulted for complex infectious cases. Since this observational point-prevalence study of patient files was performed in the context of a quality improvement project, approval of the medical ethics committee was not required. Patient data were entered anonymously in the study database.

### Data collection

#### Point prevalence survey

Four point-prevalence surveys (PPS) of in-hospital antimicrobial use^,^ were performed on four consecutive Mondays, in which all hospitalized patients were analyzed for the use of antimicrobials on that day. PPS quickly give an accessible insight in antibiotic use, and they are used in many studies for identifying targets for improvement [6–10]. The PPS were carried out according to the ECDC technical document on PPS [11]. All antimicrobial prescriptions on the concerning Mondays were included. The intensive care units (neonatal, pediatric and adult) were excluded. Files of admitted patients were manually screened for the use of antimicrobials. On Tuesday, we obtained a list from the computerized medication order entry system of the clinical pharmacy department containing all antimicrobial prescriptions of the previous Monday to confirm and complete the acquired data. We included antiviral and antifungal medication. Surgical prophylaxis administered in the operating theatre was not included.

#### Classification of prophylaxis

Prescriptions were separated into two groups: prophylactic and therapeutic prescriptions. Prophylaxis was divided into three groups: medical prophylaxis, prophylaxis around non-surgical interventions (hereafter called medical intervention prophylaxis) and surgical prophylaxis given on the ward (as opposed to prescribed in the operating theatre.) Medical prophylaxis was defined as an antimicrobial prescribed for prevention of an infectious complication of a disease. Medical intervention prophylaxis was defined as an antimicrobial prescribed for preventing infectious complications of a medical intervention or procedure which due to its nature (no incision) cannot be called surgery (e.g., endoscopy or cystoscopy). Surgical prophylaxis given on the ward was defined as any antimicrobial administered on the ward preceding a surgical intervention or administered postoperatively as extended prophylaxis to prevent surgical site infections. Prophylactic antibiotics given after surgery for other reasons than preventing wound infections or surgical complications were classified as medical prophylaxis. Indications of prophylaxis were investigated by reviewing the medical records. When indications were not clear, they were more thoroughly investigated by looking at culture samples, radiology reports and the case notes of consulted specialists.

#### Protocol presence and adherence

To evaluate the appropriateness of the prescriptions, we evaluated every prophylactic prescription for the presence of a protocol and assessed the adherence to the protocol. Local antimicrobial guidelines in the Academic Medical Center in Amsterdam (AMC) can be easily found on the intranet and are based on national guidelines, with local additions and changes made according to resistance patterns in the hospital. Other specialist guidelines are also found on the intranet, albeit less organized. We searched the local protocol database for the presence of protocols concerning prophylactic antimicrobial drug use.

Since prophylaxis in neutropaenia should be discontinued when neutropaenia is over or when the patient suffers from an active infection (neutropaenic fever), we investigated whether this prophylaxis was indeed discontinued when necessary. It was also registered when there was a documented reason for deviating from the protocol, which was scored as the motivation of non-adherence. The appropriateness of these cases was judged on a case-by-case basis.

### Data analysis

To report the extent of antimicrobial prophylaxis, ‘Days on Therapy’ (DoT) are preferably used. In practice, when conducting a PPS, a DoT is equal to a prescription of any dose of any antimicrobial. Therefore, we report data as number of prescriptions. When a patient switched from intravenous to the same oral antibiotic on the day of the PPS, this was interpreted as one DoT, but when a patient on a specific day switched from one antibiotic to another, it was interpreted as two DoT, since at least one dose of two different antimicrobials were given that day.

The extent of prophylaxis is reported as a percentage (prophylactic prescriptions/total number of prescriptions *100). The presence of a protocol was assessed for each prescription and is reported as percentage of the total number of prophylactic prescriptions. Likewise, the adherence to protocols was calculated and reported as percentage of prophylactic prescriptions with a protocol. Prophylaxis is reported by medical specialty. Some subspecialties are reported separately (e.g. haematology), in case of a large amount of prescriptions for medical prophylaxis. Since this was an explorative study, further statistical analyses were not performed.

## Results

A total of 1020 antimicrobial prescriptions were retrieved, of which 317 (31.1%) were considered prophylaxis (Table 1). Medical prophylaxis accounted for 237 prescriptions (23.2%), 22 prescriptions were prophylaxis in case of medical interventions (2.2%), and 58 (5.7%) surgical prophylaxis prescribed on a ward instead of in the operating theatre. When further differentiating into antibiotics, antifungals and antivirals, 827 of 1020 prescriptions were antibiotics. Of these, 220 (26.6%) were prophylaxis, of which 141 were medical prophylaxis (17.0% of all antibiotic prescriptions), 22 medical intervention prophylaxis (2.7%) and 57 surgical prophylaxis (6.9%). There were 96 antiviral prescriptions, of which 41 were medical prophylaxis (42.7%), while 56 of 97 antifungal prescriptions were prophylaxis (57.7%). These were most commonly prescribed in the internal medicine and haematology department.

**Table 1:** Antimicrobial prescriptions per point prevalence survey

	Total number of prescriptions	Prophylactic prescriptions (%)	Medical prophylaxis (%)	Medical intervention prophylaxis (%)	Surgical prophylaxis^a^ (%)	Antibiotic prescriptions	Antiviral prescriptions	Antifungal prescriptions
PPS1	229	60 (26.2%)	40 (17.5%)	5 (2.2%)	15 (6.8%)	188	21	20
PPS2	261	80 (30.7%)	67 (25.7%)	4 (1.5%)	9 (3.4%)	204	33	24
PPS3	259	81 (31.3%)	62 (23.9%)	5 (1.9%)	14 (5.4%)	210	22	27
PPS4	271	96 (35.4%)	68 (25.1%)	8 (3.0%)	20 (7.4%)	225	20	26
Total	1020	317 (31.1%)	237 (23.2%)	22 (2.2%)	58 (5.7%)	827	96	97

Divided in antibiotic, antiviral and antifungal prescriptions *PPS* point prevalence survey. ^a^ surgical prophylaxis given on a ward

When looking at protocol adherence in case of antibiotic prophylaxis (Table 2), for medical prophylaxis a protocol was present in 125 of 141 prescriptions (88.7%), and 118 prescriptions were given according to these protocols (94.4%). A motivation of non-adherence, in which the choice for that particular prophylaxis was specified in the patient record, was present in only one of seven cases. In the other six cases, there was no information on the reason for the deviation from the protocol. On the haematology department, prophylaxis was stopped when a patient developed fever or was not neutropaenic anymore, while in the children’s oncology department there were four cases in which prophylaxis was continued during fever.

**Table 2:** Prophylactic antibiotic prescriptions

	Prescriptions (n)	Presence of protocol (%)	According to protocol (%)	Motivation of non-adherence (%)
Medical prophylaxis (%)	141	125 (88.7%)	118 (94.4%)	1/7 (14.3%)
Medical intervention prophylaxis	22	13 (59.1%)	12 (92.3%)	0/1
Surgical prophylaxis^a^	57	42 (73.7%)	41 (97.6%)	0/1
Total	220	180 (81.8%)	171 (95.0%)	1/9 (11.1%)

^**a**^Surgical prophylaxis given on a ward

For prophylaxis in case of medical interventions, a protocol was present in 13 of 22 cases (59.1%), which was adhered to in 12 cases (92.3%). Surgical prophylaxis given on a ward had a protocol presence of 73.7% and adherence rate of 97.6%.

Antiviral and antifungal prophylaxis were primarily prescribed as medical prophylaxis (100% and 98.2%), had a protocol available in 100% and 98.2% and an adherence rate to these protocols of 95.1% and 96.3% respectively (not shown in table).

Thus, a protocol was present in 276 of 317 prophylactic antimicrobial prescriptions (87.1%), and 262 of these were according to this protocol (94.9%). Fourteen of 276 prescriptions (5.1%) where a protocol was available deviated from that protocol, which was motivated in only one case. Therefore, in 13 prescriptions (4.7%) the protocol was not followed, without documentation. In five cases, prophylaxis was motivated despite absence of a protocol.

Table 3 shows the number of antibiotic prescriptions per medical subspecialty. Haematology was the top prescriber, counting more prescriptions than no. 2 (paediatric oncology) and 3 (general internal medicine) combined. In the surgical department, both urology and orthopedic surgery were relatively large prescribers, mainly for medical intervention prophylaxis and (extended) surgical prophylaxis prescribed on the ward. These tables also show the number of prescriptions in which a protocol was present and followed. Of note, the department of hematology had a 100% guideline presence for medical prophylaxis and orthopaedic surgery had 100% protocol presence for extended surgical prophylaxis prescribed on the ward. The identified indications for prophylaxis are summarized in Table 4.

**Table 3:** Prophylactic antibiotic prescriptions per specialty/ward

Internal medicine	Prescriptions (n)	Presence of protocol (%)	According to protocol (%)	Motivation of non-adherence	Medical prophylaxis	Medical intervention prophylaxis	Surgical prophylaxis
Internal medicine	25	23 (92.0%)	23 (100.0%)	-	23	0	2
Haematology	62	62 (100%)	62 (100%)	-	62	0	0
Pulmonology	3	3 (100%)	2 (66.7%)	0/1	1	0	2
Cardiology	11	11 (100%)	10 (90.9%)	0/1	1	10	0
Gastro-enterology	8	8 (100%)	8 (100%)	-	5	3	0
Psychiatry	2*	2 (100%)	2 (100%)	-	2	0	0
Surgery							
General surgery	7	2 (28.6%)	2 (100.0%)	-	6	0	1
Oral & maxillofacial surgery	1	0 (0%)	0 (0%)	-	1	0	0
ENT-surgery	10	9 (90.0%	9 (100.0%)	-	2	1	7
Neurosurgery	5	3 (60.0%)	3 (100.0%)	-	1	1	3
Gynaecology and obstetrics	3	2 (66.7%)	2 (100.0%)	-	0	0	3
Orthopaedic surgery	17	17 (100%)	17 (100%)	-	2	0	15
Thoracic surgery	1	0 (0%)	0 (0%)	-	0	0	1
Urology	23	8 (34.8%)	8 (100.0%)	-	0	5	18
Paediatrics							
General paediatrics	16	5 (31.3%)	3 (60.0%)	0/2	10	1	5
Paediatric oncology	26	25 (96.2%)	20 (80.0%)	1/5	25	1	0

**Table 4:** Indications for prophylaxis per specialty

Internal medicine	Indication	Protocol
Haematology	1. Long-term neutropaenia/selective decontamination of the digestive tract 2. Antiviral prophylaxis after chemotherapy	1. Yes 2. Yes
Internal medicine	1. PCP and CMV prophylaxis after kidney transplantation 2. Voiding cysto-urethrography after kidney transplantation 3. Other PCP-prophylaxis	1. Yes, protocol on intranet 2. No protocol on intranet 3. Yes/no, depends on indication.
Gastro-enterology	1. ERCP 2. Esophaegeal varix haemorrhage 3. Immunosuppression for inflammatory bowel disease 4. Spontaneous bacterial peritonitis prophylaxis	1. Yes 2. Yes 3. Yes 4. Yes
Pulmonology	1. Exacerbation COPD	1. Yes
Cardiology	1. Mitraclip 2. ICD/pacemaker implantation 3. TAVI-procedure 4. Endocarditis prophylaxis	1. Yes 2. Yes 3. Yes 4. Yes
Pediatrics		
General paediatrics (including subspecialties)	1. NUSS-procedure 2. Adenotonsillectomy 3. Recurrent urinary tract infections 4. ERCP 5. HIV-prophylaxis in newborns 6. Prophylactic antibiotics in cystic fibrosis	1. Yes 2. No 3. Yes 4. No 5. Yes 6. No
Paediatric oncology	1. Long-term neutropaenia/selective decontamination of the digestive tract 2. PCP-prophylaxis	1. Yes 2. Yes
Surgery		
General surgery	1. Stoma reversal 2. Chronic anastomotic leakage 3. Non-surgical prophylaxis after Whipple operation 4. After amputation for osteomyelitis	1. No protocol 2. No protocol 3. No protocol 4. No protocol
Urology	1. TURP 2. Ureterorenoscopy 3. Double-J-catheter replacement 4. Percutaneous kidney stone removal 5. Laparoscopic/open nephrectomy 6. Cryo-ablation of tumor 7. Bricker bladder surgery	No internal protocols, prescriptions seem based on international guidelines, no documentation. Standard surgical prophylaxis according to general surgical guidelines.
Orthopaedic surgery	1. Use of osteosynthesis/joint replacement material	1. Yes
Gynaecology and obstetrics	1. Third and fourth degree rupture 2. Inguinal lymph node dissection	1. Protocol on intranet, contradicted by local antibiotics guideline 2. No mention of antibiotics in protocol
Oral & maxillofacial surgery	1. Skull fracture	1. Antibiotics mentioned in protocol, no specifics
ENT-surgery	1. Surgical prophylaxis 2. After DRAF-procedure	1. Yes 2. No protocol, expert opinion
Thoracic surgery	1. TAAA-procedure	2. No protocol
Neurosurgery	1. Deep Brain Stimulation placement 2. CNS Leakage	1. Yes 2. No protocol

Table 5 shows the antibiotics prescribed. The most prescribed prophylactic antibiotics were trimethoprim/sulfamethoxazole (32.3% of total, mainly Pneumocystis jiroveci pneumonia (PCP)-prophylaxis and urological prophylaxis), first-generation cephalosporins (21.8%, mainly surgical prophylaxis prescribed on the ward) and fluoroquinolones (20.4%, mainly selective decontamination of the digestive tract in neutropaenic patients).

**Table 5:** List of prescribed prophylactic antibiotics per antibiotic class

	Number of prescriptions (% of total)	Medical prophylaxis	Medical interventional prophylaxis	Surgical prophylaxis
Trimethoprim/sulfamethoxazole	71 (32.3%)	59	3	9
First generation cephalosporins	48 (21.8%)	3	7	38
Fluoroquinolones	45 (20.4%)	42	2	1
Small spectrum penicillins	19 (8.6%)	15	3	1
Amoxicillin/clavulanic acid	9 (4.1%)	7	1	1
Third generation cephalosporins	5 (2.3%)	1	3	1
Broad spectrum penicillins	4 (1.8%)	1	0	3
Colistine	4 (1.8%)	4	0	0
Macrolides	3 (1.4%)	3	0	0
Aminoglycosides	3 (1.4%)	1	1	1
Metronidazole	2 (0.9%)	1	0	1
Clindamycin	2 (0.9%)	1	1	0
Nitrofurantoine	2 (0.9%)	2	0	0
Meropenem	1 (0.4%)	0	1	0
Second generation cephalosporins	1 (0.5%)	0	0	1
Trimethoprim	1 (0.5%)	1	0	0
Total	220 (100%)	141	22	57

## Discussion

In this study, we investigated the extent, indications and appropriateness of antimicrobial prophylaxis that was given outside the operating theatre in a tertiary care teaching hospital. A substantial part (31.1%) of all antimicrobial prescriptions (including antivirals and antifungals) on the wards was for prophylaxis. Almost a quarter of all antibiotic prescriptions concerned prophylaxis. And around 50% of antiviral and antifungal prescriptions. For most antibiotic medical prophylaxis there was a protocol and adherence to these protocols was high. For antibiotic prophylaxis around non-surgical interventions and for surgical prophylaxis given on the ward a protocol was often not available, but if present, adherence again was high. Antivirals and antifungals were with a few exceptions given according to a protocol.

The percentage of about a third of all antimicrobial prescriptions administered outside the operating theatre being prophylaxis is in line with reports from other tertiary care hospitals in the Netherlands. A study from Rotterdam reported that 34.4% of hospitalized patients used prophylactic antibiotics, but this included patients using surgical prophylaxis administered in the operating theatre [9]. A study from the Radboud University hospital in the Netherlands reported a percentage of 19.1% for medical prophylaxis, but only antibiotics were investigated [10]_._ The annual report on antibiotic use from 50 mainly non-academic hospitals in the Netherlands (Nethmap 2015) reports a percentage of medical prophylaxis of 12.7% [12]. This may indicate a lower proportion of medical prophylaxis in secondary care centers. None of these reports contain indications, information on departments or data on the appropriateness of the prescriptions. A multi-center audit of antibiotic use in France, including both university and non-university hospitals, reported a percentage of medical prophylaxis of 11.2%, comparable to the number found in the Nethmap study [13].

The adherence rate to protocols for medical antibiotic prophylaxis (94.4%) is high. Most audits of therapeutic antibiotic use report lower percentages of guideline adherence [14]. In the aforementioned study from Rotterdam, therapy was considered appropriate in 70.7%. The high guideline adherence rate in prophylaxis may be explained by the fact that prophylaxis is more easily protocolized than therapy.

This study has several strengths. It was done on four different time points, involving all wards, and the manual identification of antimicrobial prescriptions was validated by a list from the computerized medication order entry system. This implies that it is unlikely that prescriptions were overlooked. Additionally, it provides an overview of the specific indications, existence of and adherence to protocols, and differences between medical specialties.

Limitations are that some indications might have been missed, for instance if certain procedures are never performed on Mondays. The radiology department documents antimicrobial use in a different system, and their patients do not show up in the medical records. This might have led to an underestimation of prophylaxis for medical interventions. Also, documentation of indications was sometimes poor. We finally decided to exclude the intensive care unit in our study, despite the common use of prophylactic selective decontamination of the gastrointestinal tract, because our intensive care unit was working with a different medication ordering system.

From our study, the most important target for improvement is the absence of protocols for antibiotic prophylaxis during medical interventions. However, considering the multitude of interventions and the complex cases in a tertiary care hospital, some cases are to be left to the expertise of the treating clinician. Surgery in children with specific congenital malformations and complex surgical patients are examples where antimicrobial prophylaxis might be indicated, but where standardization will be very difficult. Appropriateness in these cases is therefore difficult to assess. An additional problem is that despite the presence of protocols, the evidence for some indications is limited. For urology, evidence is available for transurethral resection of the prostate and prostate biopsy, but for all other procedures it is unclear whether prophylaxis is necessary [15, 16]. Also for orthopedic surgery, where the use of extended surgical prophylaxis (prescribed on the ward) in revision surgery is clearly protocolized, the evidence is lacking [17]. For most cardiologic procedures, there are no randomized controlled trials on prophylactic antibiotic use. Even for PCP prophylaxis, despite its wide-spread use, there are three different dosing schemes in use and unclear evidence on which one is preferable [18]_._

Further studies should focus on the role of antimicrobial prophylaxis given outside the operating theatre in non-tertiary care centers. It is possible that in non-university hospitals, with their different case-mix, e.g., less haematological and solid organ transplant patients and less complex surgical procedures, prophylaxis will be a smaller part of total antimicrobial use. Additionally, the outpatient clinic would be interesting to investigate, as many prophylactic antimicrobial prescriptions may be initiated or continued there.

## Conclusions

A third of antimicrobial prescriptions prescribed outside the operating theatre concerns prophylaxis. Whereas in our hospital antimicrobial prophylaxis outside the operating theatre in general turned out not to be an important target for improving antimicrobial use, in particular prophylaxis for non-surgical interventions and surgical prophylaxis given on the ward deserves attention from each antimicrobial stewardship team, in particular the availability of protocols for these indications.

## Abbreviations

DOT: Days on therapy
ECDC: European Center for Disease Control
PCP: Pneumocystic jiroveci pneumonia
PPS: Point prevalence survey
RCT: Randomized controlled trial
